# Does Mutation Rate Depend on Itself?

**DOI:** 10.1371/journal.pbio.0060052

**Published:** 2008-02-26

**Authors:** Charles F Baer

## Abstract

Recent evidence suggests that mutation rates are fitness-dependent, broadening our view of the impacts of mutation on the genetic health of populations.

Many a research paper, textbook chapter, and grant proposal has begun with the phrase “Mutation is the ultimate source of genetic variation.” Implicit in this phrase is the assumption that genetic variation is required for evolution. Without mutation, evolution would not be possible, and life itself could never have arisen in the first place. However, there is overwhelming evidence that the great majority of mutations with detectable effects are harmful [1–3]. Deleterious mutations are the price we living organisms pay for the ability to evolve.

Deleterious mutations are known or thought to influence a wide variety of biological phenomena (see the special issue of Genetica (1998) for a comprehensive account), the most notable of which is sexual reproduction. To make a very long story short, there are several reasons that asexually reproducing taxa are expected to have a short-term evolutionary advantage over sexually reproducing taxa. Specifically, the fact that a gene is passed to all the offspring of an asexual individual and to only half the offspring of a sexually reproducing individual provides a 2-fold fitness advantage to a new mutation that confers asexual reproduction when it arises in a population of sexual organisms. This 2-fold difference in fitness is known as the “2-fold cost of meiosis” [4]. However, a wealth of empirical evidence suggests that asexual reproduction is an evolutionary dead end, at least for eukaryotic taxa [5].

Understanding why and how sexual taxa have managed to consistently overcome the 2-fold cost has probably absorbed more intellectual energy than any other single problem in evolutionary biology. Many arguments have been advanced to explain the prevalence of sexual reproduction [4,6], but the most widely accepted arguments invoke deleterious mutations as a primary cause [7–9]. The *sine qua non* of sex is genetic recombination, and deleterious mutations are more readily removed from a population in the presence of recombination than in its absence. A large body of theory predicts that, all else equal, the greater the mutation rate, the greater the probability that sexual reproduction will be favored. However, recent research by Aneil Agrawal [10,11] calls that conclusion into question.

A key property shared by all of the underlying models is the assumption that the mutation rate is constant, although it has long been recognized that mutation rates vary between and even within taxa [1,12]. However, there is intriguing evidence not only that the mutation rate is variable within groups but that the variation in mutation rate is correlated with fitness, such that low-fitness individuals have higher mutation rates. A correlation between fitness and mutation rate could have two (not mutually exclusive) underlying causes, one adaptive and one not adaptive. The “adaptive mutation” scenario has been influential in the world of microbial genetics, following the observation of Cairns and Foster [13] that Escherichia coli have higher mutation rates when starved (reviewed in [14]). The basic idea of adaptive mutation is that under normal conditions, low mutation rate is favored by selection because most mutations are deleterious. However, in a very poor environment, death is certain in the absence of a beneficial mutation that confers high fitness in that environment. Individuals with high mutation rates are more likely to “find” that beneficial mutation. Thus, natural selection will favor inducible mutation rates, which are low under normal conditions but greatly increased under stressful (i.e., low-fitness) conditions. Adaptive mutation remains controversial, but there is evidence from E. coli that the stress-induced mutation rate differs consistently with certain ecological circumstances [15].

Importantly, the adaptive mutation scenario is only plausible in taxa that reproduce primarily asexually, because recombination breaks up the association between the alleles that influence mutation rate and alleles that influence fitness [16,17]. Another, perhaps more important possibility is that individuals in poor physiological condition have higher mutation rates for reasons having nothing to do with the possibility of generating a lucky beneficial mutation. Assuring the fidelity of DNA replication is metabolically costly and involves the products of many dozens or hundreds of genes [18]. Individuals in poor condition will have fewer resources to devote to genomic surveillance, leading to the possibility that individuals in poor condition will suffer an increased mutation rate.

Poor physiological condition may occur for two fundamental reasons, environment and/or genes. A simple example of the former is genetically identical plants raised under variable conditions of moisture: plants that are watered will be in better condition than those that are not. An example of the latter is harder to contrive, but it has been shown that under identical environmental conditions, there is genetic variation for fitness. Variation in fitness could be due either to variation in the number of deleterious alleles an individual carries in its genome or variation in the effects on fitness, or both. If individuals carrying more deleterious alleles tend to be in poor condition and if individuals in poor condition tend to have higher mutation rates, the existence of a positive-feedback process is suggested, which leads to an upwardly spiraling mutation rate and downwardly spiraling fitness.

## Fitness-Dependent Mutation Rate: Theoretical Predictions

A useful way of quantifying population-genetic phenomena is in terms of the “genetic load,” the reduction in fitness of a population of interest relative to a population composed solely of the most-fit genotype. Of particular interest is the genetic load at mutation-selection balance (MSB), the point at which the input of genetic variation from mutation is exactly balanced by the removal of deleterious mutant alleles by natural selection. A classic, perhaps surprising result is that the genetic load at equilibrium in an infinitely large population is determined solely by the genomic deleterious mutation rate (*U*), which is assumed to be constant, and is independent of the strength of selection [19]. At MSB, mean fitness Ŵ is approximately e*^−U^* and the genetic load is 1 − e*^−U^*; this result holds for both sexual and asexual populations, in the absence of nonadditive effects [20].

A functional relationship between fitness and mutation rate complicates the situation. The first theoretical treatment of fitness-dependent mutation rates was given by Agrawal [10] for the infinite-population case. His model allowed mutation rate to vary over some range of possible values (*U*_MIN_ to *U*_MAX_), with individuals in the best-possible condition mutating at rate *U*_MIN_ and those in the worst-possible condition (other than dead) at *U*_MAX_. The functional relationship between fitness and *U* is assumed monotonic but not necessarily linear, and the fitness effects of mutations are assumed multiplicative across loci.

**Figure 1:**
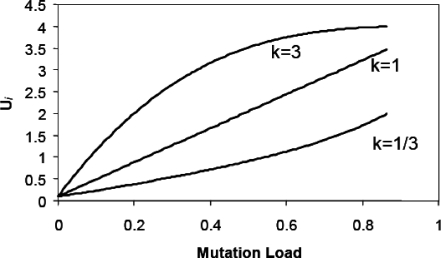
Graph of Mutation Rate (*U*) against Mutation Load Plot of *U* as a function of mutation load, scaled relative to that of the least-loaded genotype (*U*_MIN_ = 0). *U_i_* = *U*_MAX_− (*U*_MAX_ −*U*_MIN_) W_i_^k^. Load = 1 − *w_i_* where *w_i_* is relative fitness of a genotype with *i* deleterious mutations; *w_max_* = *w*_0_ = 1. Note: this figure is slightly different from the analogous figure in [10] in which mutation rate is plotted against fitness rather than load.

Two important proximate results emerge from Agrawal's analysis. First, for an asexual population, mean fitness at equilibrium is a function of the mutation rate of the genotype with the fewest deleterious alleles (i.e., the smallest genetic load). If the most-fit genotype has zero deleterious mutations, Ŵ_asex_ ≈e^−*U*_MIN_^, even though most genotypes in the population may have many more than zero mutations. Second, the equilibrium fitness of a sexual population depends critically on the nature of the functional relationship between mutation rate and fitness (Figure 1). If the mutation rate is a concave downward (decelerating) function of genetic load, then Ŵ_sex_ will approach that expected at the maximum mutation rate (*U*_MAX_). Conversely, if the mutation rate is a concave upward (accelerating) function of genetic load, Ŵ_sex_ will be much closer to that expected at the minimum possible mutation rate (*U*_MIN_).

This analysis points to a key difference in the equilibrium genetic load in sexual and asexual populations under constant and fitness-dependent mutation rates. If the mutation rate is constant, then sexual and asexual populations are expected to have the same genetic load at equilibrium. In contrast, if the mutation rate is fitness-dependent, the expected fitness in an asexual population at equilibrium will be that of the most-fit genotype, while in a sexual population, the equilibrium fitness will be less than that of the most-fit genotype, perhaps much less. Thus, surprisingly, the “cost of sex” may in fact be much greater than 2-fold.

## Fitness-Dependent Mutation Rate: Empirical Evidence

Although condition-dependent, inducible increases in mutation rate are well-documented in microbes, including yeast [21] and the unicellular alga Chlamydomonas [22], there is scant evidence from multicellular eukaryotes. A recent study by Ávila et al. [23] showed that the rate of decay of viability and the rate of increase of genetic variance in a stock of Drosophila melanogaster that had been allowed to accumulate mutations for 160 generations increased 2.5-fold over the ancestral stock. That result is consistent with an increase in the mutation rate with genetic load (as claimed by the authors in the title of the article), but it is also consistent with an increase in the average deleterious effect of a new mutation with increasing genetic load, i.e., “synergistic” epistasis [20].

In this issue of *PLoS Biology*, Agrawal and Wang report a study designed to determine if the mutation rate is condition-dependent in D. melanogaster without the ambiguity associated with classical mutation accumulation studies [11]. Their experiment takes advantage of the fact that DNA repair does not occur post-meiotically in male D. melanogaster, but DNA damage carried in sperm can be repaired post-fertilization in D. melanogaster embryos via maternal repair enzymes. Manipulating female condition could alter maternal repair processes. There are multiple maternal repair pathways, some of which are error prone and some of which are not. The error-prone pathways are believed to be less metabolically costly. Agrawal and Wang hypothesized that females in poor condition would preferentially use the less-costly error-prone mechanisms, whereas females in good condition would preferentially use the more-costly high-fidelity mechanisms. Condition was manipulated by diet; genetically identical females were fed high- or low-quality diets and fertilized with sperm that had been subject to mutagenesis. The relative frequency of recessive lethal alleles was 30% greater on paternally derived X chromosomes that had passed through low-condition females rather than high-condition females.

Do these results prove that the mutation rate is condition-dependent? Not quite. It remains to be shown that the spontaneous mutation rate is condition-dependent. However, if the efficacy of DNA repair is in general condition-dependent, a lack of condition-dependence of the spontaneous mutation rate would require there be a compensatory reduction in the probability that DNA damage occurs. Nevertheless, this study provides the strongest evidence yet for condition-dependent mutation rate in a multicellular eukaryote.

## Broader Implications

Condition- (i.e., fitness) dependent mutation leads to some interesting possibilities for the long-term genetic health of populations, including our own species. For example, it has been argued that modern technology (e.g., sewage treatment, eyeglasses, etc.) has led to the relaxation of selection against mildly deleterious mutations in the developed world, leading to a build-up of genetic load [24,25]. If the mutation rate is self-dependent, future generations may be burdened with an ever-growing genetic load. If the somatic and germ-line mutation rates are correlated, such a build-up of genetic load could be expected to lead to an increase in cancer and other diseases resulting from somatic mutation. (Less often invoked is the possibility that the improved physiological condition of modern humans will act to *reduce* the mutation load).

A second interesting possibility is that condition-dependent mutation could, in effect, render temporary increases in mutagenesis due to environmental causes permanent. Many anthropogenic factors are known to be mutagenic, not only to humans but to many other organisms. If the mutation rate is condition-dependent, a short-term increase in the input of mutation due to (say) a mutagenic pollutant could lead to a long-term increase in the mutation rate, and thus in the genetic load. Similarly, genetic drift allows the fixation of slightly deleterious alleles in small populations. With condition-dependent mutation rate, a temporary bottleneck in population size that results in the increase in frequency of deleterious alleles could lead to an effectively permanent increase in the mutation rate.
